# Functional comparison of SP6 RNA polymerase and T7 RNA polymerase

**DOI:** 10.1371/journal.pone.0351567

**Published:** 2026-06-22

**Authors:** Julia Gutbrod, Svenja Hehn, Antonia Bangnowski, Eleanor Fritz, Andreas Marx

**Affiliations:** Department of Chemistry, University of Konstanz, Konstanz Research School Chemical Biology, Konstanz, Germany; Chung-Ang University, KOREA, REPUBLIC OF

## Abstract

RNA-based therapeutics have emerged as a powerful class of drugs, highlighted by the rapid development and success of COVID-19 vaccines. Therapeutic RNA synthesis relies on *in vitro* transcription (IVT), most commonly using bacteriophage RNA polymerases (RNAP) such as T7 RNAP and SP6 RNAP. However, efficient incorporation of modified nucleotides and the reduction of double-stranded RNA (dsRNA) by-products to improve pharmacokinetic properties and reduce immunostimulatory effects remain major challenges. While T7 RNAP has been extensively engineered to expand substrate tolerance and reduce dsRNA formation, engineering efforts for SP6 RNAP remain comparatively limited, and a direct functional comparison between the two enzymes is lacking. Here, we systematically compared wild-type (wt) T7 and SP6 RNAP with respect to nucleotide analogue incorporation and dsRNA formation. We evaluated engineered variants of both polymerases for their ability to incorporate the modified nucleotides 2′‑F‑UTP and 2′‑O‑methyl‑UTP during IVT. In general, T7 RNAP displayed higher yields than SP6 RNAP for long RNA transcripts and a T7 RNAP variant demonstrated the highest acceptance of 2’‑F‑UTP. However, when using 2’‑OMe‑UTP in IVT, only a SP6 RNAP enabled synthesis of a ~ 760 nt long transcript. Moreover, the assessment of dsRNA formation with both wild-type polymerases revealed that SP6 RNAP produced substantially less dsRNA than T7 RNAP during IVT. Together, these results highlight distinct and complementary strengths of T7 and SP6 RNAP. While the SP6 RNAP FA variant showed particular promise for the synthesis of mRNA containing bulky nucleotide modifications, the reduced dsRNA formation observed for SP6 RNAP wt suggests an additional advantage for improving RNA quality.

## Introduction

*In vitro* transcribed RNA has become a central component of modern RNA therapeutics, with applications ranging from mRNA vaccines [1,2] and cancer vaccines [3,4] to protein replacement [5,6] and gene regulation [7,8]. Research has spiked, especially on the development of mRNA vaccines, which have proven to be a powerful tool against viral pathogens like SARS‑CoV‑2 [1,2]. Viral antigens are encoded on the mRNA and trigger an immune response upon translation in the human cells. The rapid and flexible design of RNA-based drugs has driven increasing demand for robust and scalable transcription systems. Single-subunit bacteriophage RNA polymerases are widely used for IVT due to their simplicity and high productivity [9]. However, the biochemical properties of these enzymes strongly influence RNA yield, stability, and purity.

To enhance stability, improve translation efficiency, and reduce immunostimulatory effects, therapeutic RNA is frequently modified with nucleotide analogues [10,11]. Chemical modifications such as 2′‑*O*‑methyl (2′‑OMe), 2′‑*O*‑methoxyethyl (2′‑MOE), and 2′‑fluoro (2′‑F) have been shown to increase nuclease resistance, improve pharmacokinetics, and attenuate innate immune activation, particularly in siRNA-based applications [12–14]. However, the efficient incorporation of such chemically modified nucleotides by RNA polymerases is often limited, representing a major bottleneck in the production of therapeutic RNA. Beyond the limited substrate flexibility of RNA polymerases, the formation of immunogenic double-stranded RNA (dsRNA) represents a major challenge in the production of therapeutic RNA. Promoter-independent transcription and 3′‑extension of run-off transcripts can result in the synthesis of complementary RNA strands that anneal to the primary transcript as antisense RNA [15]. In addition, abortive RNA fragments and the 3′ ends of full-length transcripts can serve as primers for complementary RNA synthesis, ultimately leading to dsRNA formation [16]. Such dsRNA is recognized in the cytoplasm as a viral signature, triggering innate immune activation and suppression of protein synthesis [16]. Consequently, minimizing dsRNA formation by RNA polymerases during IVT is a key objective for improving the quality and safety of therapeutic RNA.

The RNA polymerase from bacteriophage T7 is the most widely used enzyme for *in vitro* synthesis of therapeutic RNA, in particular, mRNA [17]. This ~99 kDa single-subunit polymerase produces high RNA yields and can be readily expressed and purified [18,19]. However, its stringent substrate specificity limits the efficient incorporation of chemically modified nucleotides. In addition, T7 RNAP exhibits limited RNA-dependent activity, promoter-independent transcription, and untemplated 3′‑extension, all of which contribute to the formation of dsRNA [20–22]. To address these limitations, T7 RNAP has been extensively engineered for more than three decades [23]. Directed evolution, as well as rational and computational design strategies, have focused on expanding substrate tolerance, improving thermostability, and reducing by-product formation such as dsRNA and abortive transcripts [21,24–26]. Leveraging detailed structural insights obtained by X‑ray crystallography [27–29], rational design enabled the generation of the T7 RNAP F variant (Y639F) [23]. Mutation of Y639, a residue responsible for discrimination between ribose and deoxyribose, permits efficient incorporation of 2′‑NH_2_- and 2′‑F‑modified nucleotides [30]. Additional targeting of H784, which directly interacts with the 2′‑OH group of the ribose, yielded the Y639F/H784A double mutant (FA), enabling incorporation of bulkier modifications such as 2′‑OMe and 2′‑N_3_ nucleotides during mRNA synthesis [25]. Beyond substrate specificity, rational engineering of T7 RNAP has also reduced dsRNA formation arising from abortive transcription and loop-back transcription [21], or promoter-independent transcription [22]. Moreover, high-temperature IVT using thermostable T7 RNAP variants has been shown to suppress dsRNA formation [31].

An alternative to T7 RNAP is SP6 RNAP, a ~ 96 kDa single-subunit RNA polymerase derived from bacteriophage SP6, which infects *Salmonella typhimurium* LT2 [32,33]. Although SP6 RNAP is capable of producing high yields of mRNA, it is far less frequently used in therapeutic RNA production [34]. This limited adoption can partly be attributed to the lack of detailed structural information. For T7 RNAP, multiple crystal structures have been reported, including initiation and elongation complexes as well as an intermediate complex containing a 7-nt RNA oligonucleotide [27,29,35,36]. In contrast, no experimentally determined structure of SP6 RNAP is currently available. Comparative studies of T7, T3, and SP6 RNAPs have revealed similar misincorporation properties, suggesting a conserved catalytic mechanism among these polymerases [37]. However, mechanistic differences that may affect substrate specificity, processivity, or dsRNA formation remain poorly understood. While the Y631F mutant of SP6 RNAP, analogous to the T7 RNAP F variant, has been reported in a patent, systematic engineering efforts targeting SP6 RNAP are scarce [38]. As a result, the potential of SP6 RNAP, and engineered SP6 variants, for the synthesis of modified RNA and for reduced dsRNA formation remains largely unexplored.

Despite extensive engineering of T7 RNAP, a systematic comparison of T7 and SP6 RNAP has not been performed. To evaluate whether SP6 RNAP represents a viable alternative to T7 RNAP for the synthesis of therapeutic RNA, we directly compared wild-type (wt) and engineered variants of both polymerases with respect to nucleotide analogue incorporation and dsRNA by-product formation. AlphaFold2-based modeling [39] of SP6 RNAP was performed to gain structural insights and facilitate comparison with the well-characterized T7 RNAP structure. Guided by structure- and sequence homology to T7 RNAP, we generated the SP6 RNAP Y631F mutant (SP6 RNAP F) as well as the previously unreported double mutant Y631F/H779A (SP6 RNAP FA). The transcriptional activity of these variants was compared to the corresponding T7 RNAP F (Y639F) and FA (Y639F/H784A) mutants using the nucleotide analogues 2′‑F‑UTP and 2′‑OMe‑UTP. In addition, we evaluated and compared the dsRNA formation by T7 RNAP wt and SP6 RNAP wt.

## Results and discussion

### Structural comparison of T7 and SP6 RNAP and generation of SP6 RNAP mutants

**Fig 1:**
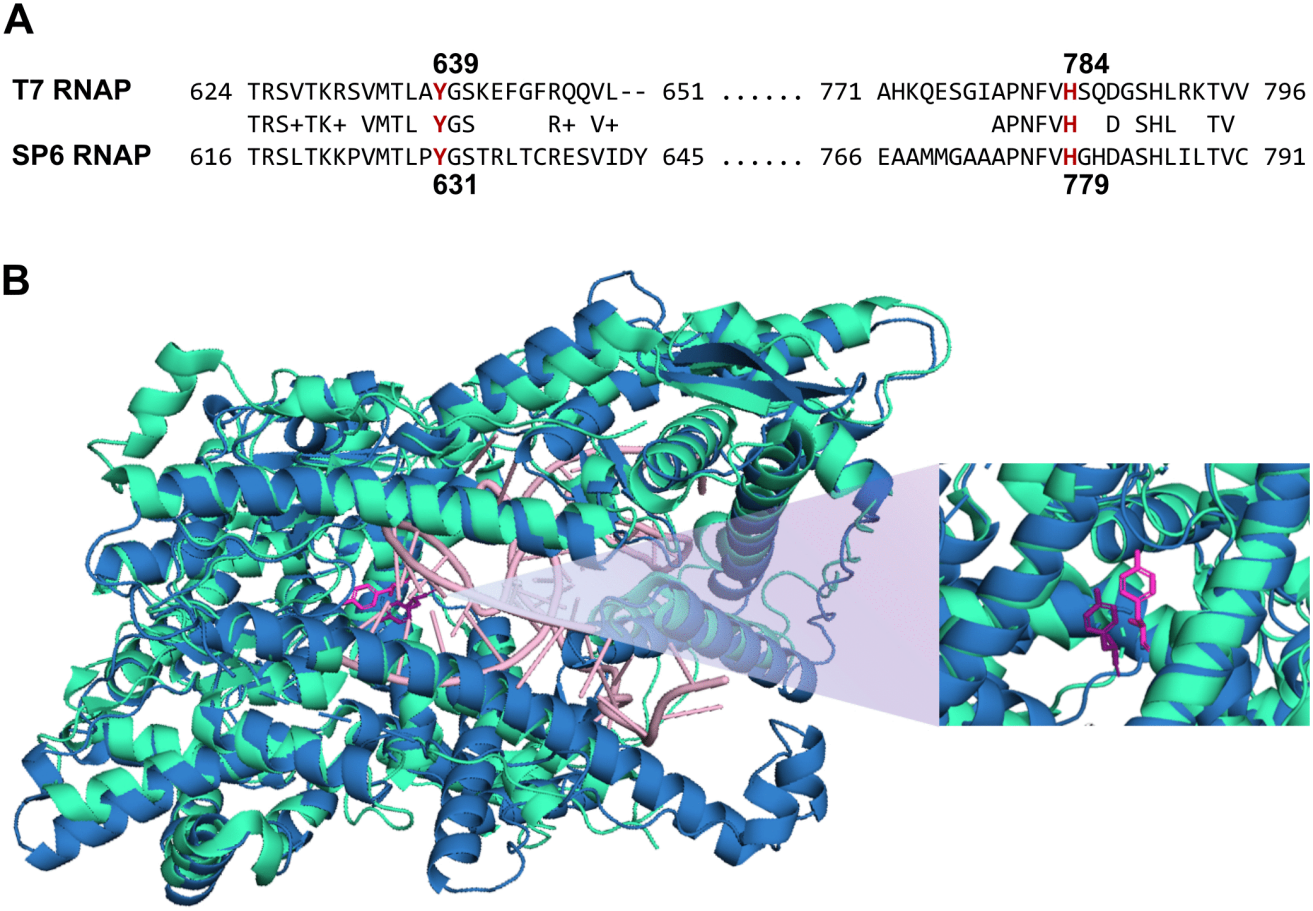
Sequence alignment and structural comparison of T7 RNAP and SP6 RNAP. **(A)** A sequence alignment of T7 RNAP and SP6 RNAP shows conservation of residues within the catalytic pocket. Tyrosine 639 (T7 RNAP) and 631 (SP6 RNAP) as well as Histidine 784 (T7 RNAP) and 779 (SP6 RNAP) are highlighted in red. **(B)** Alignment of a T7 RNAP intermediate complex crystal structure (PDB: 3E2E) (green) containing a DNA template and a 7 nt RNA oligo (rose), with the SP6 RNAP structure predicted by AlphaFold2 (blue). Residue Y639 (T7 RNAP, purple) and Y631 (SP6 RNAP, pink), as well as residues H784 (T7 RNAP, black) and H779 (SP6 RNAP, grey) are highlighted.

The lack of structural data available for SP6 RNAP limits direct structure-guided engineering of this enzyme. To address this limitation, the amino acid sequence of SP6 RNAP was submitted to AlphaFold2 for structure prediction. Among the five generated models, the structure with the highest predicted confidence was selected to perform structural alignment with the T7 RNAP intermediate complex (PDB: 3E2E) [36]. Sequence alignment reveals conservation of key residues such as the above described tyrosine and histidine, found in the active site of both polymerases (Fig 1A). Superposition of the SP6 RNAP (blue) AlphaFold2 structure with T7 RNAP 3E2E (green) also demonstrated a high degree of structural similarity with residues Y639/Y631 (purple/pink) as well as H784/H779 (black/grey) occupying similar positions within the catalytic site (Fig 1B). These observations suggested that introducing corresponding mutations in SP6 RNAP could elicit effects comparable to those previously described for the T7 RNAP Y639F single mutant and Y639F/H784A double mutant, potentially enabling incorporation of modified nucleotides.

Based on these insights, SP6 RNAP variants Y631F (F) and Y631F/H779A (FA) were generated by site-directed mutagenesis using the wild-type SP6 RNAP gene as template. All mutations were confirmed by sanger sequencing. Wild-type and mutant SP6 RNAPs were heterologously expressed in *E. coli*, containing an N-terminal His-tag. Proteins were purified in two steps, using affinity chromatography and ion exchange chromatography and analyzed by SDS-PAGE to confirm purity. T7 RNAP variants were used as previously prepared in our group and purified via affinity chromatography [40].

### Activity and substrate tolerance of T7 RNAP, SP6 RNAP and their mutants on a short template

**Fig 2:**
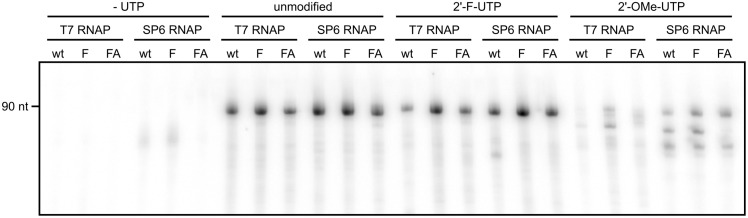
Radioactive sequencing PAGE assay with 2‘-*O*-modified UTP analogues. The activity of T7 RNAP wt and its mutants F (Y639F) and FA (Y639F, H784A) was compared to the activity of SP6 RNAP and its mutants F (Y631F) and FA (Y631F, H779A). IVT reactions were performed with a template resulting in a 89 nt (T7 RNAP)/ 88 nt (SP6 RNAP) template, in absence of UTP (- UTP), with UTP (unmodified) or with 2‘‑F‑UTP or 2‘‑OMe‑UTP. Transcripts were labeled by the incorporation of [α^32^P] GTP and analyzed via denaturing PAGE.

To initially evaluate and compare the transcription activity of T7 RNAP wt and its F and FA variants with SP6 RNAP wt and the corresponding F and FA variants, IVT assays were performed using a short DNA template. These reactions yielded RNA transcripts of 89 nt for T7 RNAP and 88 nt for SP6 RNAP. Based on preferences reported in the literature, the transcription start sequence GGGAGA was used for T7 RNAP [41] and GAAGA for SP6 RNAP [34], while the remaining template sequence was identical for both enzymes. To assess incorporation of modified nucleotides, UTP was substituted with either 2’‑F‑UTP or 2’‑OMe‑UTP. Negative control reactions were performed in the absence of UTP. RNA transcripts were radioactively labeled by inclusion of [α^32^P]GTP and analyzed via denaturing PAGE (Fig 2). All reactions were carried out under matched conditions with identical templates downstream of the polymerase-specific promoter, identical NTP pools, buffer composition, temperature and reaction time, so that any differences in promoter recognition, initiation efficiency, abortive cycling or modified-nucleotide incorporation rate between T7 and SP6 RNAP are reflected directly in the integrated readouts of full-length transcript yield and length distribution. Endpoint analysis under such matched conditions has been established as the appropriate readout for comparing IVT performance, including by-product profiles, in studies of T7 RNAP variants aimed at therapeutic mRNA production [21,26,31,37].

All polymerase variants exhibited transcription activity with natural nucleotides (unmodified) and were able to synthesize the full-length transcript. When omitting UTP from the reactions (-UTP), reactions performed with SP6 RNAP wt and SP6 RNAP F show diffuse band pattern upon the PAGE analysis, indicating the synthesis of shorter RNA species. This likely reflects misincorporation of other nucleotides at positions normally occupied by uridine, however no polymerase was able to synthesize a full-length transcript in the absence of UTP. When UTP was replaced with 2’‑F‑UTP, transcription activity of both T7 RNAP wt and SP6 RNAP wt was reduced, whereas the F and FA variants of both polymerases largely retained their activity. Notably, T7 RNAP F and SP6 RNAP F exhibited similar efficiencies and were the most active enzymes under these conditions. In T7 RNAP, residue Y639 plays a critical role in recognizing the hydrogen-bonding capacity of the 2’‑substituent of incoming nucleotides. Substitution of tyrosine with phenylalanine abolishes this hydrogen-bonding interaction and creates additional space within the catalytic pocket, thereby facilitating incorporation of nucleotides carrying 2’‑modifications [30]. The results shown in Fig 2 suggest an analogous role for residue Y631 in SP6 RNAP, as mutation to phenylalanine similarly enhances incorporation of 2’‑F‑UTP. In reactions containing the bulkier 2’‑OMe‑UTP, transcriptional activity of T7 RNAP was strongly reduced. PAGE analysis revealed a smear indicative of abortive transcription for T7 RNAP wt and T7 RNAP FA, and neither enzyme produced a detectable amount of the full-length 89 nt transcript. Only the T7 RNAP F variant synthesized a small amount of full-length RNA under these conditions. In contrast, SP6 RNAP wt as well as both the F and FA variants were able to produce the full-length 88 nt transcript, albeit with reduced efficiency. Notably transcription activity of SP6 RNAP F and FA using 2’‑OMe-UTP was similar and superior to wild-type activity. The presence of truncated products indicated premature termination, potentially caused by polymerase stalling during incorporation of two consecutive UTP analogues at positions 76 and 77 of the transcript. Notably, formation of these abortive products was reduced in reactions performed with the SP6 RNAP FA variant. Structural studies of T7 RNAP have shown that histidine 784 directly interacts with the incoming nucleotide [29]. Mutation of this residue to alanine enlarges the active site and reduces steric hindrance, facilitating incorporation of bulky nucleotide analogues such as 2’‑OMe and 2’‑N_3_ [25]. Although enhanced activity of the T7 RNAP FA variant was not observed in the present assay, the corresponding substitution in SP6 RNAP (H779A) may contribute to improved activity in the presence of 2’‑OMe‑UTP. This effect could explain the reduced formation of abortive transcripts observed for the SP6 RNAP FA variant. Transcription activity is a key determinant for efficient synthesis of therapeutic RNA. Extensive downstream purification is required to remove abortive RNA fragments to ensure product safety and efficacy, often at the expense of yield. Therefore, identifying RNAP variants that incorporate modified nucleotides while maintaining activity represents a crucial step toward optimizing enzymatic mRNA production.

### Activity and substrate tolerance of T7 RNAP, SP6 RNAP and their mutants on an extended template

**Fig 3:**
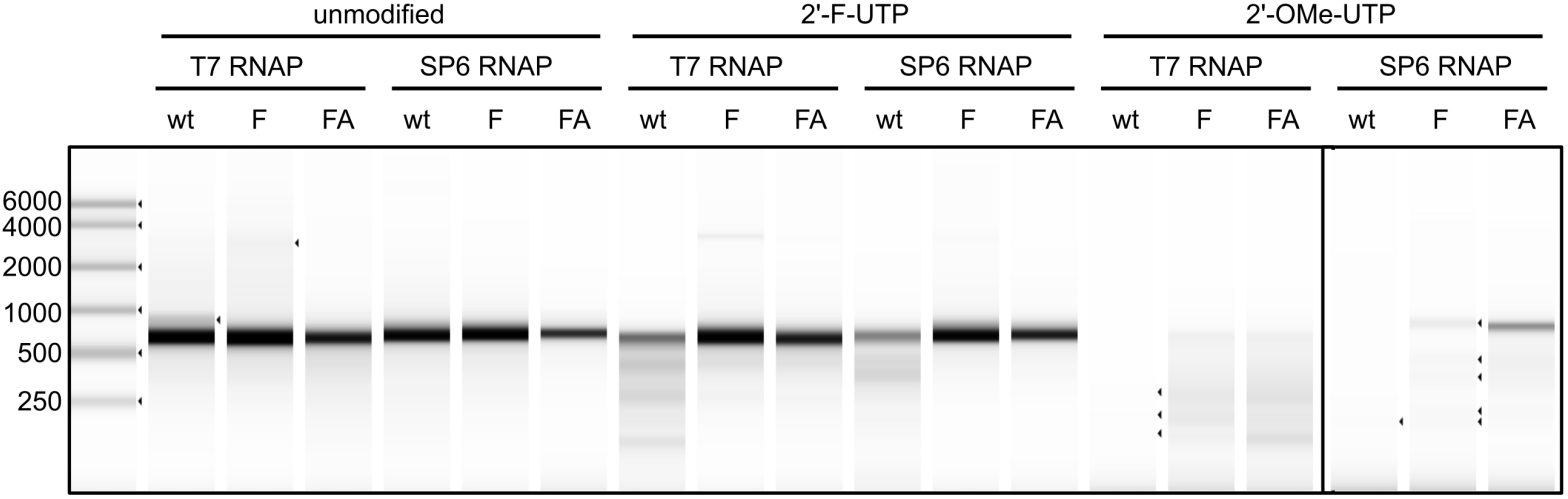
TapeStation assay with 2‘-*O*-modified UTP analogues. The activity of T7 RNAP wt and its mutants F (Y639F) and FA (Y639F, H784A) was compared to the activity of SP6 RNAP and its mutants F (Y631F) and FA (Y631F, H779A) in *in vitro* transcription reactions. An EGFP coding template was transcribed, resulting in a ~ 760 nt long RNA. Reactions were performed in absence of UTP (- UTP), with UTP (unmodified) or with 2‘‑F‑UTP or 2‘‑OMe‑UTP. Transcripts in the reactions were analyzed using the Agilent TapeStation system. RNA concentrations in the IVT reactions, determined by the TapeStation, are given in µg/µL.

While transcription of short templates, such as those used in the sequencing PAGE assays described above, provides a useful initial assessment of polymerase activity and nucleotide analogue incorporation, evaluation of polymerase activity during synthesis of long transcripts requires additional analysis. To assess the activity of SP6 RNAP variants during transcription of extended RNA sequences in the presence of modified nucleotides, IVT reactions were performed using a linearized DNA template encoding EGFP. This template yielded a full-length RNA transcript of approximately 760 nt. RNA product formation was analyzed using the Agilent TapeStation system (Fig 3).

All polymerase variants were able to synthesize full-length transcripts using unmodified UTP. However, RNA concentrations were generally higher in reactions performed with T7 RNAP. For both T7 and SP6 RNAP, the FA variant displayed the lowest activity with natural nucleotides. Consistent with the results obtained using short templates, both F and FA variants of T7 and SP6 RNAP retained transcriptional activity when UTP was substituted with 2’‑F‑UTP. In contrast, activity of both wild-type enzymes was strongly reduced under these conditions, accompanied by the formation of shorter RNA species, most prominently for T7 RNAP wt, suggesting increased abortive transcription. When 2’‑OMe-UTP was used in IVT reactions, neither wild-type polymerase produced detectable RNA. T7 RNAP F and FA generated smeared signals in the TapeStation gel images, indicating the presence of truncated RNA products, but showed no or only minimal synthesis of full-length transcripts. Among all enzymes tested, SP6 RNAP FA was the only polymerase that produced a clearly resolved full-length 760 nt transcript with 2’‑OMe‑UTP, despite its overall lower total RNA concentration; this lower concentration is consistent with reduced abortive cycling rather than impaired full-length synthesis, as discussed below. The reduced total RNA concentration observed for SP6 RNAP FA may reflect a lower abundance of abortive transcripts, as indicated by the reduced diffuse band pattern compared to T7 RNAP FA. In T7 RNAP wt and SP6 RNAP wt, steric hindrance imposed by the bulky 2’-OMe modification within the catalytic pocket likely resulted in complete loss of activity. In contrast, for T7 RNAP F and FA as well as SP6 RNAP F, steric constraints did not abolish activity entirely but may have induced polymerase stalling during elongation, leading to premature termination and accumulation of abortive transcripts. Consequently, although T7 RNAP FA remained catalytically active, it lacked sufficient activity to generate the 760 nt RNA using 2’‑OMe‑UTP. In SP6 RNAP FA, overall activity was also reduced; however, as discussed above, the combined Y631F and H779A substitutions likely expanded the catalytic pocket sufficiently to permit synthesis of full-length RNA containing 2’‑OMe‑UTP to a limited extent.

Overall, these findings show that rational engineering of SP6 RNAP can broaden the range of nucleotide chemistry accessible by IVT. In particular, the SP6 RNAP FA variant enables synthesis of mRNA containing bulky 2’‑modified nucleotides that are poorly tolerated by wild-type polymerases. Importantly, these results emphasize SP6 RNAP as a promising alternative to T7 RNAP. The retained activity of SP6 RNAP FA in the presence of 2’‑OMe‑UTP underscores its suitability for RNA synthesis and highlights the potential of targeted active-site engineering to improve RNA polymerase performance. Further structural research and engineering of this variant could enhance enzyme stability and activity, thereby allowing efficient synthesis of even longer RNA transcripts, necessary for mRNA therapeutics.

Taken together, Figs 2 and 3 define a clear polymerase-by-nucleotide preference profile across both template lengths. With unmodified UTP, both enzymes synthesize full-length transcripts, but T7 RNAP and its variants give consistently higher yields on the 760 nt template. With 2’‑F‑UTP, the F variants of both polymerases are the most active, with T7 RNAP F showing the highest acceptance of this modification. With the bulkier 2’‑OMe‑UTP, the polymerase ranking inverts: only SP6 RNAP variants produce detectable full-length 760 nt RNA, with the SP6 RNAP FA variant uniquely yielding a clearly resolved full-length band, whereas T7 RNAP variants are limited to truncated products under identical conditions. Thus, T7 RNAP F is the preferred enzyme for 2’‑F-modified mRNA, while SP6 RNAP FA is the preferred enzyme for the incorporation of bulkier 2’‑OMe modifications into long transcripts.

### dsRNA formation by wild-type polymerases

**Fig 4:**
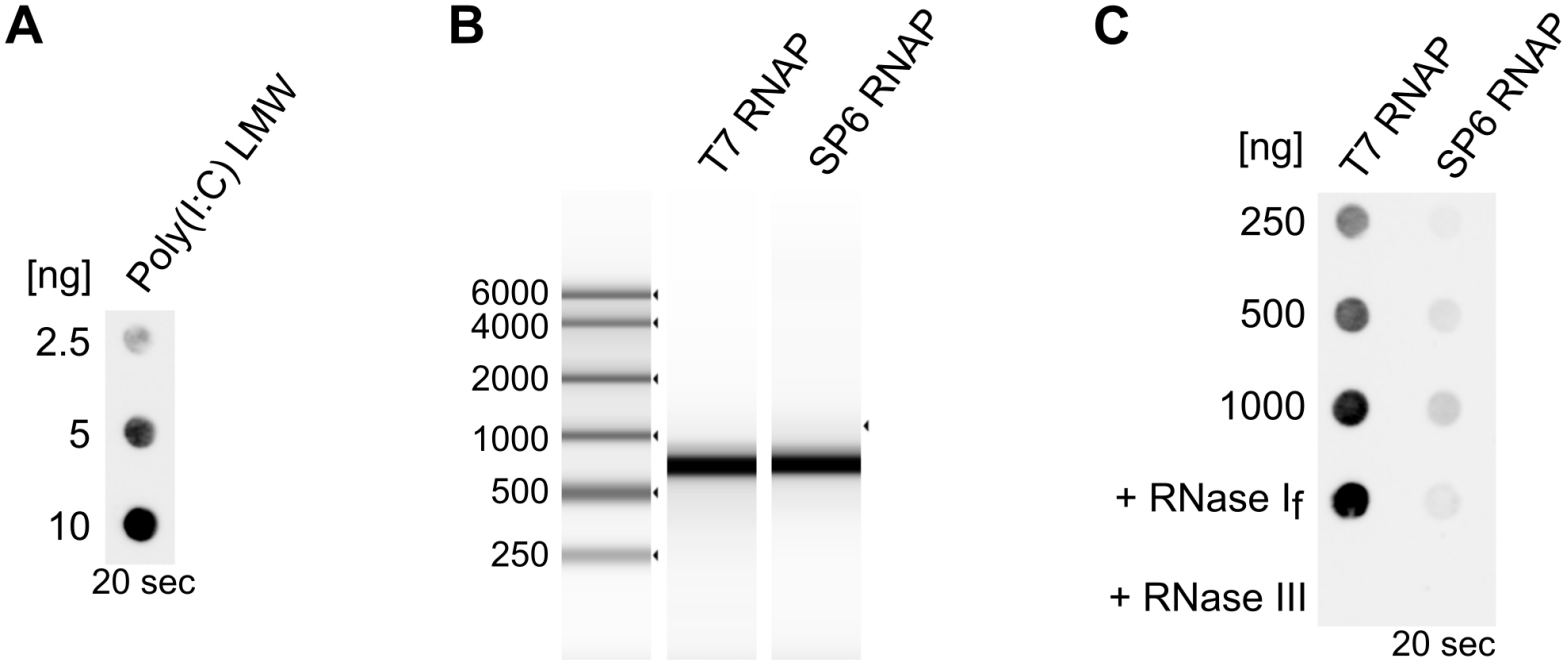
Evaluation of dsRNA formation in IVT reactions using the J2 antibody. **(A)** Different amounts of Poly(I:C) LMW were applied to the Cytiva Nytran™ SuperCharge membrane as positive control. Poly(I:C) was detected using the J2 antibody (Scicons) and chemiluminescent readout at an exposure time of 20 sec. **(B)** Transcripts in IVT reactions with either T7 RNAP or SP6 RNAP were analyzed using the Agilent TapeStation system. Concentrations of RNA in the reactions are given in µg/µL. (C) dsRNA content in the reactions from (B) was evaluated by applying different amounts of RNA to the Cytiva Nytran™ SuperCharge membrane. 1000 ng of RNA were either treated with RNase If or with ShortCut® RNase III. dsRNA was detected using the J2 antibody (Scicons) and chemiluminescent readout at an exposure time of 20 sec.

To evaluate and compare dsRNA formation by T7 RNAP wt and SP6 RNAP wt we performed a dot-blot assay and detected dsRNA directly in IVT reactions using the dsRNA specific J2 antibody (Fig 4). We first validated the detection method, confirming concentration-dependent binding of the dsRNA-specific J2 antibody by applying increasing amounts of low-molecular-weight poly(I:C) to a positively charged nylon membrane. Following incubation with the J2 antibody and a peroxidase-coupled secondary antibody, chemiluminescent detection revealed a clear concentration-dependent signal, confirming specific recognition of the double-stranded polymer (Fig 4A). Next we performed IVT reactions using the above utilized EGFP template with either T7 RNAP wt or SP6 RNAP wt. TapeStation analysis demonstrated that both enzymes produced full-length RNA transcripts of comparable yield and high purity (Fig 4B). Based on the RNA concentrations determined by TapeStation, defined amounts of RNA were applied to the membrane, and dsRNA content was detected using the J2 antibody. Across all tested RNA amounts, dot intensities obtained from T7 RNAP-derived samples were markedly stronger than those from SP6 RNAP-derived samples, indicating substantially higher dsRNA formation by T7 RNAP under identical conditions. To confirm the nature of the detected RNA species, 1,000 ng of RNA from each sample was treated with either the ssRNA-specific RNase I_f_ or the dsRNA-specific ShortCut® RNase III prior to membrane application. RNase III treatment completely abolished the J2 signal, whereas RNase If treatment had no effect, confirming that the detected signal originated from dsRNA and demonstrating the specificity of the J2 antibody (Fig 4C).

The observation that SP6 RNAP produces significantly lower levels of dsRNA in our assay highlights its potential as a valuable enzyme for *in vitro* RNA synthesis. Future structural and mechanistic studies may elucidate the molecular basis underlying the reduced dsRNA production observed for SP6 RNAP. Moreover, targeted engineering strategies, such as improving enzyme stability or transcriptional fidelity, analogous to approaches described for T7 RNAP [21,22,31], could further reduce dsRNA formation and enhance RNA quality.

## Conclusion

In this study, we performed a systematic comparison of T7 and SP6 RNAP and their engineered variants with respect to nucleotide analogue incorporation and dsRNA by-product formation. While T7 RNAP remains the most widely used enzyme for IVT, our results demonstrate that SP6 RNAP represents a viable and, in some respects, advantageous alternative for the synthesis of modified RNA. Rationally designed mutations targeting conserved active-site residues enabled direct comparison of homologous F and FA variants in both polymerases. Substitution of the conserved tyrosine residue to phenylalanine enhanced incorporation of 2′‑F‑modified nucleotides in both T7 and SP6 RNAP. Notably, SP6 RNAP FA, with the additional histidine-to-alanine substitution, uniquely retained sufficient activity to synthesize full-length RNA in the presence of 2′‑OMe‑UTP, whereas the corresponding T7 RNAP variant predominantly produced truncated transcripts. Beyond substrate tolerance, we directly compared dsRNA formation by wild-type enzymes and found that SP6 RNAP produced substantially lower levels of dsRNA than T7 RNAP under identical transcription conditions, despite comparable overall RNA yields. Given the immunogenic nature of dsRNA, this property represents a significant advantage for synthesis of therapeutic RNA, as it diminishes the need for extensive downstream purification and lowers the risk of unintended innate immune activation *in vivo*. Taken together, our results establish SP6 RNAP as an underexplored platform for IVT. Its reduced dsRNA formation and favorable performance with modified nucleotides, particularly in engineered variants, position SP6 RNAP as a promising alternative to T7 RNAP for the synthesis of high-quality therapeutic RNA.

Building on the complementary substrate preferences uncovered here, namely T7 RNAP for 2’‑F‑modified RNA, SP6 RNAP FA for 2’‑OMe‑modified long RNA, and SP6 RNAP wt for low-dsRNA synthesis, a logical next step will be to explore whether these strengths can be combined within a single workflow. One avenue is mixed-polymerase IVT, in which T7 and SP6 RNAP are used in parallel reactions and the products are pooled, or, more ambitiously, in a single reaction where promoter orthogonality is exploited to assign distinct templates to each enzyme. Such studies will require dedicated experimental designs to deconvolve the contribution of each polymerase to yield, modified-nucleotide incorporation and dsRNA formation, for example, through promoter-specific sequence tags or differential isotopic labelling. Together with further structure-guided engineering of SP6 RNAP, such approaches may enable hybrid IVT platforms that combine the high yields of T7 RNAP with the favorable purity and modified-nucleotide tolerance demonstrated here for SP6 RNAP.

## Materials and methods

### Generation of SP6 RNAP mutants F and FA

Point mutations at positions Y631 and H779 were successively introduced into the SP6 RNAP wt polymerase gene carried on the pET21b vector using site-directed mutagenesis. *E. coli* XL10-Gold cells were transformed to identify successfully mutated variants. Colonies were screened by Sanger sequencing and plasmids containing the desired mutation were used to transform BL21 DE3 for protein expression. The polymerase variants were expressed at 25 °C over night and purified via affinity and ion exchange chromatography on an Äkta pure™ protein purification system (Cytiva). For affinity chromatography, polymerase bound to the column (HisTrap Crude FF) via N-terminal polyhistidine tag (6x His) was eluted with a linear imidazole gradient from 10 mM to 250 mM in buffer B (buffer A: 50 mM Tris‑HCl pH 7.5, 500 mM NaCl; buffer B: buffer A + 250 mM imidazole). Fractions containing SP6 RNAP were pooled and dialyzed overnight into HiTrap buffer A (50 mM Tris-HCl pH 7.5, 500 mM NaCl) for further purification via ion exchange chromatography (1 mL HiTrap Q column). Elution was carried out with a linear gradient from 50 mM to 1 M NaCl (buffer A: 25 mM Tris‑HCl pH 7.5, 50 mM NaCl, 2 mM DTT, 0.1% Triton X-100; buffer B: same as buffer A with 1 M NaCl). Polymerase-containing fractions were pooled and dialyzed overnight into 2 × storage buffer (100 mM Tris-HCl pH 7.5, 200 mM NaCl, 0.05% Triton X-100, 5 mM DTT). The purified protein was concentrated using Amicon Ultra centrifugal filter units (Millipore), and glycerol was added to a final concentration of 50%. Samples were stored at −20 °C or −80 °C for long-term storage.

### Purification of T7 RNAP variants

T7 RNAP F and FA variants as well as the wild-type were expressed in *E.coli* BL21 in the pGDR11 vector at 37 °C for 5 h. Affinity purification of His-tagged proteins was performed using cOmplete™ His-Tag Purification resin (Roche). Protein was eluted with 500 mM imidazole in buffer (50 mM Tris‑HCl (pH 8), 300 mM NaCl, 0.1% Triton X‑100) after washing of the resin with the same buffer containing 25 mM imidazole. Polymerase-containing fractions were pooled and buffer exchanged to 2 × storage buffer (100 mM Tris-HCl pH 8.0, 200 mM NaCl, 0.2% Triton X-100, 10 mM DTT, 2 mM EDTA, 1x cOmplete™ Protease-Inhibitor-Cocktail) using Amicon Ultra centrifugal filter units (Millipore). Glycerol was added to a final concentration of 50% (v/v) and samples were stored at ‑20 °C.

### Denaturing PAGE analysis

For analysis via denaturing PAGE, IVT reactions were carried out using a DNA template (0.5 µM; T7 RNAP: 5′‑GCCTCCTATAATACGACTCACTATAGGGAGACCGTCAGCTGTGCCGTCGCGCAGCACGCGCCGCCGTGGACAGAGGACTGCAGAAAATCAACCTATCCTCCTTCAGGACCAACG-3′, SP6 RNAP: 5′‑GCCTCCATTTAGGTGACACTATAGAAGACCGTCAGCTGTGCCGTCGCGCAGCACGCGCCGCCGTGGACAGAGGACTGCAGAAAATCAACCTATCCTCCTTCAGGACCAACG-3′) resulting in a 89 nt (T7 RNAP) or 88 nt (SP6 RNAP) transcript. Reactions (20 μL) contained 3 mM of each NTP, 1 × transcription buffer (40 mM Tris-HCl pH 7.5, 20 mM MgCl₂, 1 mM spermidine, 0.01% Triton X-100), 1 U/μL RNase inhibitor, 10 mM DTT, 16.65 nM [α^32^P]GTP (0.1 μL; 300 Ci/mmol, 10 mCi/mL) and 0.07 μg/μl polymerase variant. Following transcription at 37 °C for 3 h, 50 μL of PAGE stop solution (90% formamide, 50 mM EDTA, 0.01% bromophenol blue, 0.01% xylene cyanol) was added, and samples were denatured at 95 °C for 3 min. 1 µL of each reaction was loaded onto a pre-run 10% denaturing polyacrylamide gel (100 W, 30 min) and transcripts separated by applying 100 W for 45 min. Radioactively labeled RNA was visualized by phosphorimaging.

### TapeStation analysis

IVT was performed using 25 nM of HindIII linearized template containing the EGFP sequence (2960 bp, 760 nt transcript). 20 µL reactions contained 3 mM of each NTP, 1 × transcription buffer (40 mM Tris-HCl pH 7.5, 20 mM MgCl₂, 1 mM spermidine, 0.01% Triton X-100), 1 U/μL RNasin® ribonuclease inhibitor (Promega), 10 mM DTT, and 0.07 μg/μL RNA polymerase variant. Incubation was carried out at 37 °C for 2.5 h, followed by DNase I treatment (Roche, 0.2 U/µL) for 30 min at 37 °C. For analysis, crude samples were diluted 1:10 in MQ, 1 µL mixed with 5 μL RNA ScreenTape sample buffer, vortexed (IKA MS3, 2000 rpm, 1 min), denatured at 72 °C for 3 min, and chilled on ice for 2 min. After brief centrifugation, samples were analyzed using the Agilent 4150 TapeStation system.

### Evaluation of dsRNA formation

The amount of *in vitro* transcribed RNA in IVT reaction samples was determined via TapeStation. Samples were diluted in MQ to a final volume of 10 μL, containing 250 ng, 500 ng or 1000 ng of RNA. Additionally, 1000 ng of RNA were digested with either RNase I_f_ (1x NEB3 buffer, 0.5 µL RNase I_f_) or RNase III (1x short Cut RNase III buffer, 20 mM MnCl_2_, 0.5 µL RNase III) in a 10 µL reaction volume at 37 °C for 15 min, following heat inactivation at 85 °C for 5 min. Digested and undigested RNA samples were applied to a Nytran® SPC nylon membrane using the Bio-Dot apparatus (Bio-Rad) and incubated on the membrane for 30 min. The membrane was washed with TBS-T, blocked for 1 h in 5% milk in TBS-T and incubated overnight at 4 °C with the dsRNA-specific J2 antibody (1:5000 in 1% milk in TBS-T). After washing with TBS-T, a secondary HRP-conjugated goat anti-mouse IgG antibody was applied for 1 hour. The membrane was washed again and chemiluminescent detection was enabled by SuperSignal™ West Pico PLUS substrate (Thermo Scientific; mixed 1:1 Luminol/Enhancer and Stable Peroxidase Solution) and subsequent readout via ChemiDoc Imaging System (Bio-Rad).

## Supporting information

S1 File Raw images. (PDF)
