# Supplementary material for: Functional comparison of SP6 RNA polymerase and T7 RNA polymerase
Source: PLoS One. 2026 Jun 22;21(6):e0351567. doi: 10.1371/journal.pone.0351567 (PMC13286181; doi:10.1371/journal.pone.0351567)
Supplement: S1 File — (PDF) [file pone.0351567.s001.pdf]

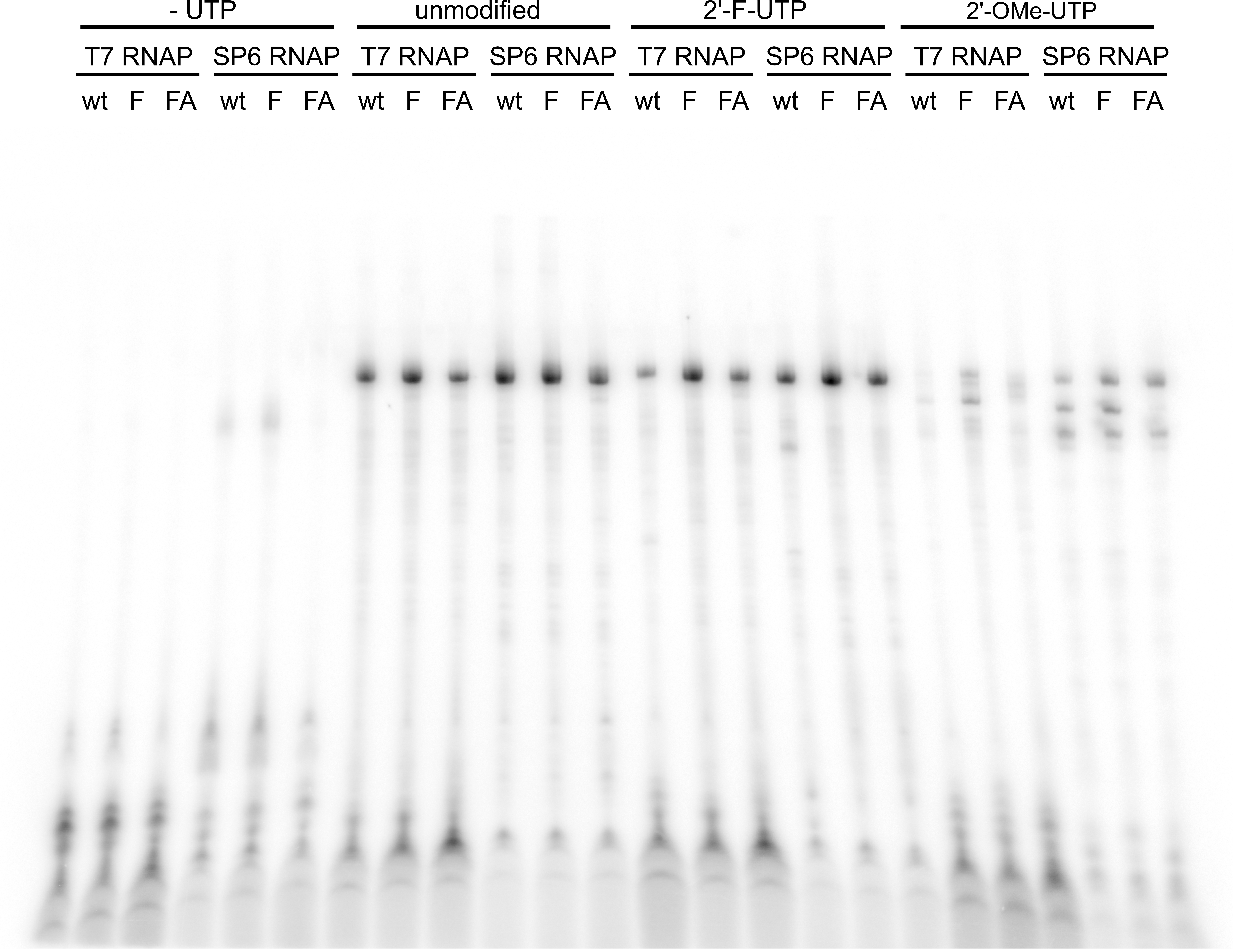

Raw image Fig 2: Phosphorimaging, 750 V

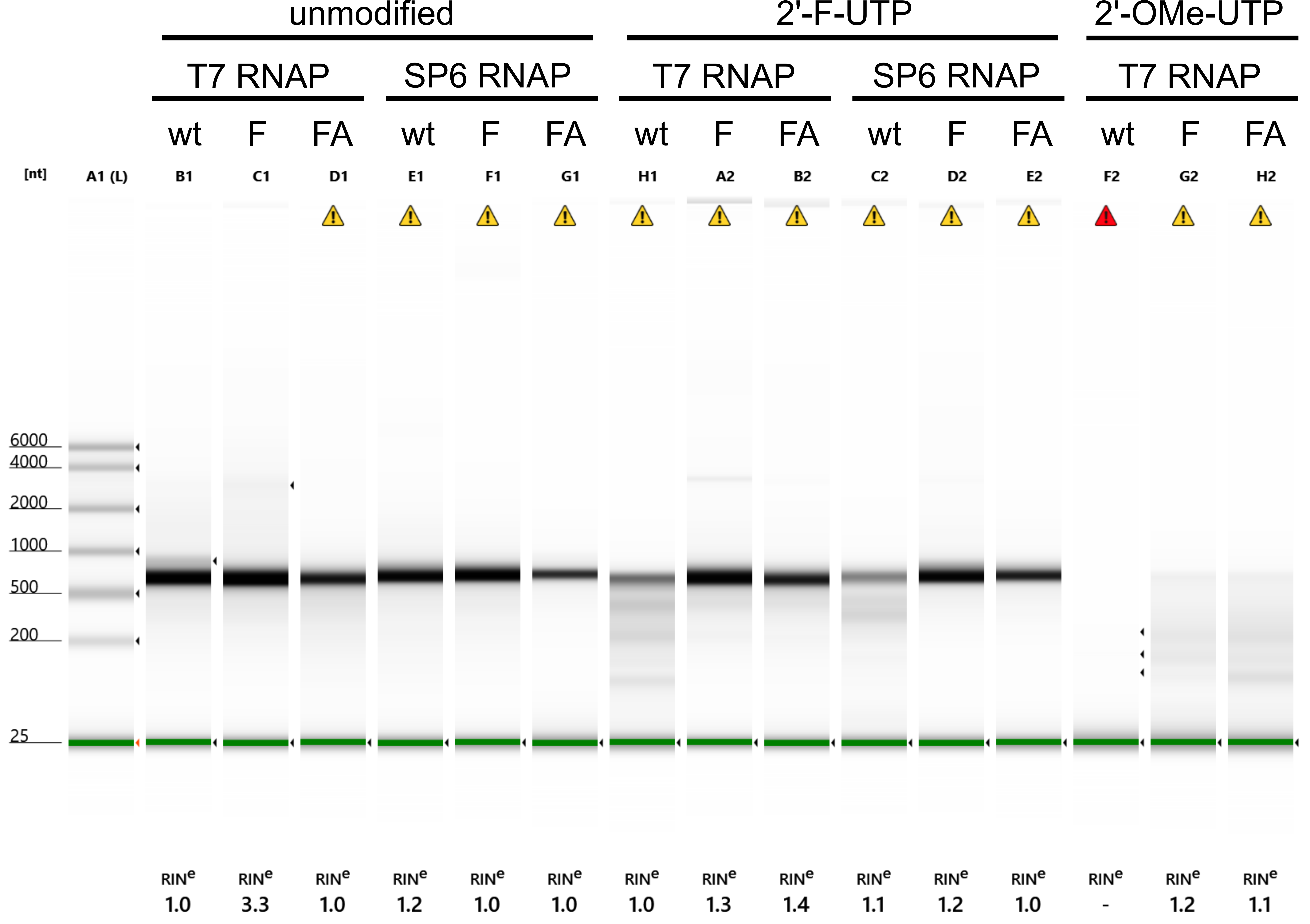

Raw image Fig 3 left: Agilent TapeStation System

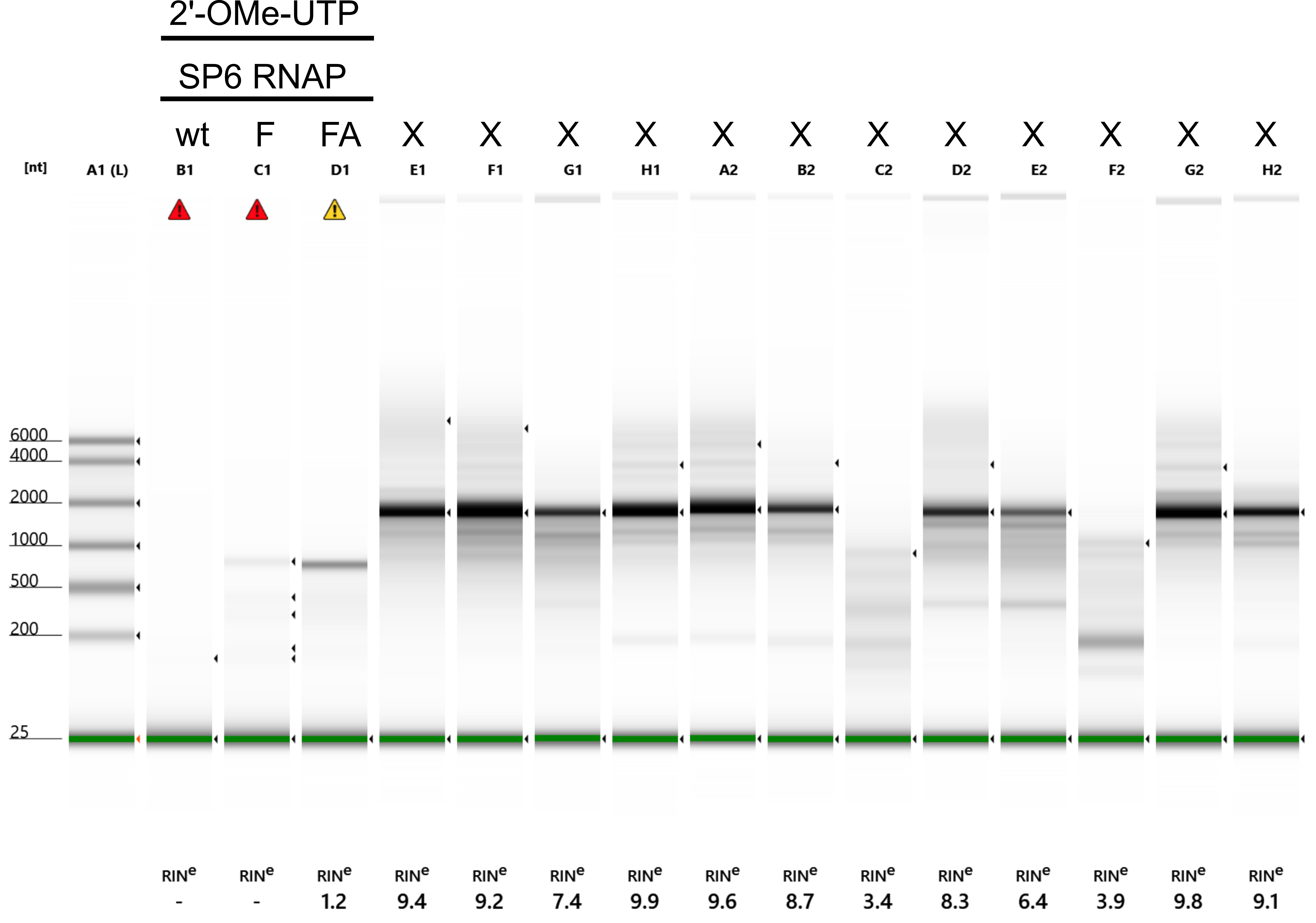

Raw image Fig 3 right: Agilent TapeStation System

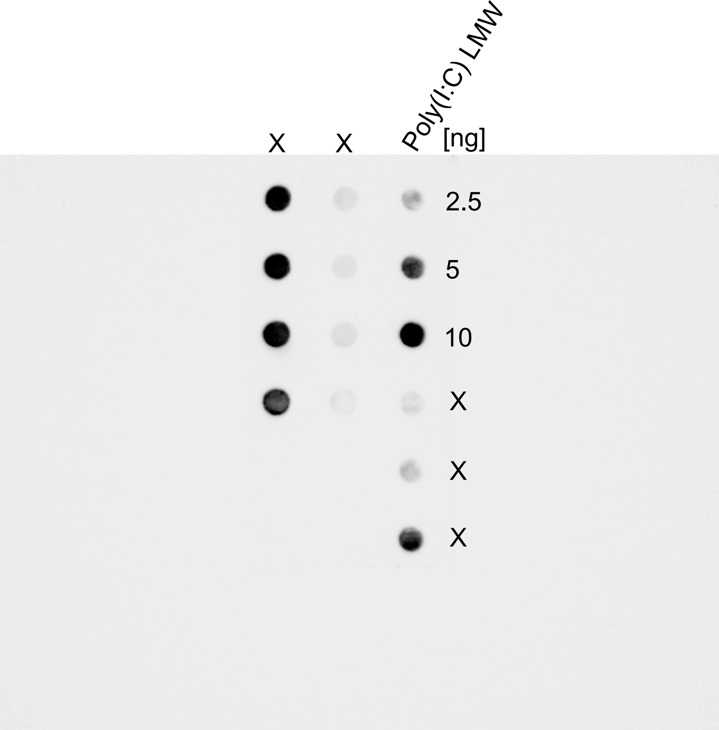

Raw image Fig 4A: Chemiluminescent detection, 425 nm, exposure time 20 sec

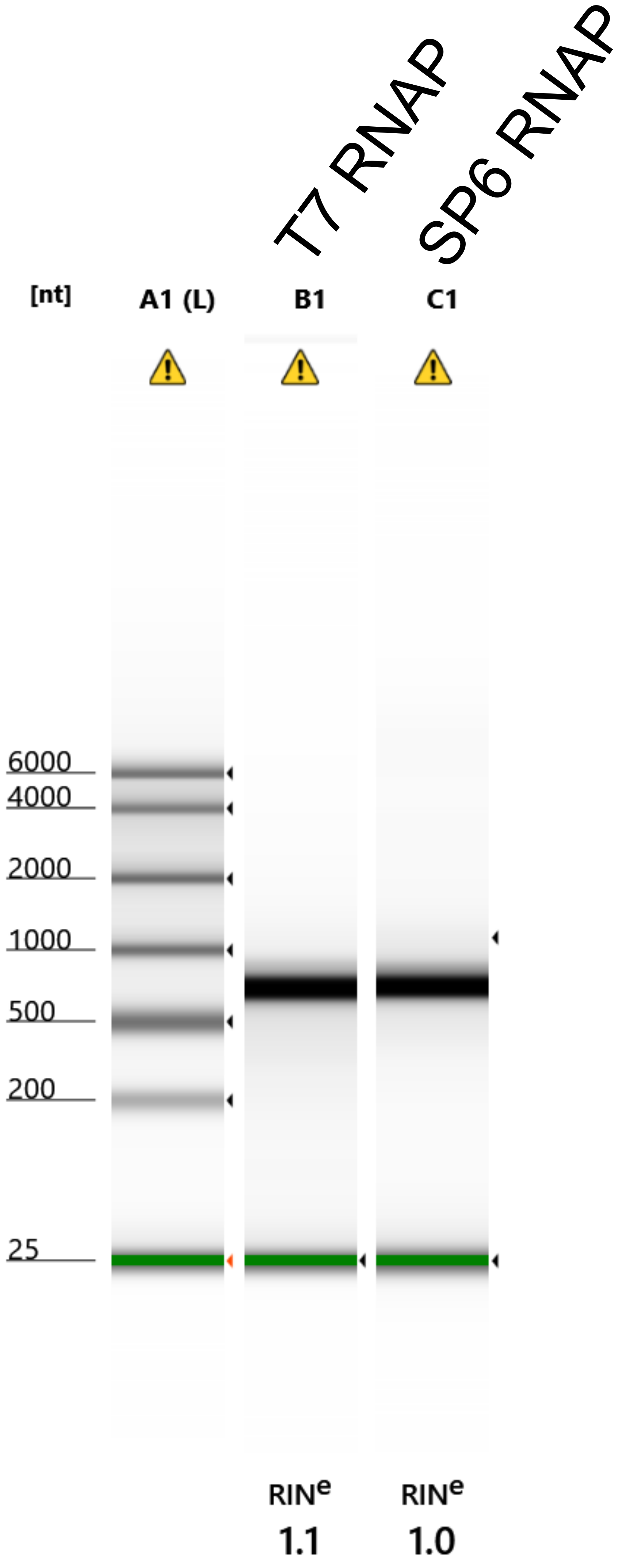

Raw image Fig 4B: Agilent Tape Station System

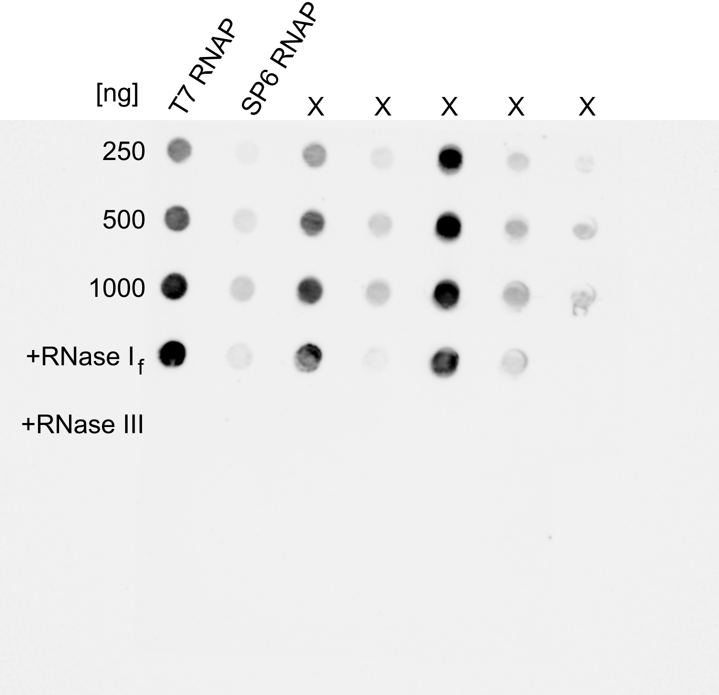

Raw image Fig 4C: Chemiluminescent detection, 425 nm,
exposure time 20 sec
