# Supplementary material for: Functional comparison of SP6 RNA polymerase and T7 RNA polymerase
Source: PLoS One. 2026 Jun 22;21(6):e0351567. doi: 10.1371/journal.pone.0351567 (PMC13286181; doi:10.1371/journal.pone.0351567)
Supplement: Attachment — Submitted filename: Response to Reviewers.pdf [file pone.0351567.s003.pdf]

RESPONSE TO THE EDITOR AND REVIEWER COMMENTS

Dear Editor and Reviewer,

We thank you for your careful evaluation of our manuscript and for the constructive feedback. We appreciate the opportunity to revise and further improve our work. We have carefully considered all comments raised by the editor and the reviewer and have revised the manuscript accordingly.

Below, we provide a detailed, point-by-point response to all comments and indicate how the manuscript has been revised.

Many thanks for your time and consideration.

Responses to Editorial Requirements

1. Compliance with PLOS ONE formatting and style requirements
We have revised the manuscript to fully comply with PLOS ONE formatting guidelines and style requirements. This includes file naming conventions, implementing sentence case for all headings, the removal of street addresses and ZIP codes as well as the removal of funding and author contributions in the manuscript. All changes are highlighted in the document "Revised Manuscript with Track Changes".

2. Role of the funders statement
We confirm the financial support from the EU Horizon 2020 programme (NEWmRNA, Grant No. 965135). We have added the following statement in the cover letter as requested: *"The funders had no role in study design, data collection and analysis, decision to publish, or preparation of the manuscript."*

3. Removal of funding information from manuscript text
All funding-related information has been removed from the manuscript file and will be included in the Funding Statement section of the submission system, in accordance with journal policy.

4. Data Availability and minimal dataset compliance
We confirm that all data required to replicate the findings of this study are provided within the manuscript and Supporting Information files.

5. Blot/gel image requirements
We have ensured that all figures comply with PLOS ONE image guidelines. Original uncropped and unadjusted blot/gel images have been provided as Supporting Information file S1_raw_images.

Response to Reviewer

Reviewer comment:

"RNA based viral therapy received attention worldwide after the success with covid pandemic and several excellent science are already being practised. From the structural point of view, the main focus need to be the stability of the double stranded RNA with modified nucleotides. A few questions come to our mind is the ease of incorporation modified nucleotides in large amount and the kinetic control of the product. Authors here use two well known viral RNAP, both single stranded proteins but no attempts are made here to follow the stability of each with the specific promoters. Some well formed transcripts are shown, but all at equilibrium. Rate of incorporation of modified nucleotides in each case is a. very important parameter which readers would like to know. Overall, I would like to see which RNAP among the two are better for which nucleotide, DNA recognition and turnover of the product. Can we use a mixture of polymerases to optimise the desired product?"

We thank the reviewer for the positive evaluation of the technical soundness and clarity of the manuscript, and for the thoughtful suggestions. Below we address each point in turn and explain how the questions raised are either already answered by the data presented in the manuscript or fall outside the scope and design of this comparative study.

1. Scope of the study: modified RNA synthesis vs. dsRNA stability

We would first like to clarify a point that we believe underlies several of the reviewer's questions. Our work does not concern modified double-stranded RNA (dsRNA) as an intended product. The two questions we address are (i) the synthesis of single-stranded modified mRNA by IVT and (ii) the formation of dsRNA as an unintended by-product of IVT, which is a well-recognized contaminant relevant to the immunogenicity of therapeutic mRNA (Mu et al., Front. Mol. Biosci. 2024; Dousis et al., Nat. Biotechnol. 2023; Kariko et al., NAR 2011). The thermodynamic stability of these dsRNA by-products as a function of nucleotide modification is therefore not a property our claims rely on, and additional stability measurements would not strengthen, refute, or refine the conclusions drawn from our data.

2. Polymerase–promoter recognition and "stability"

The reviewer asks for an explicit comparison of the stability of T7 and SP6 RNA polymerases with their respective promoters. We respectfully note that T7 and SP6 RNA polymerases are among the most extensively characterized single-subunit RNAPs: their promoter sequences are strictly orthogonal, the binding affinities of the apo and initiation complexes have been determined biochemically and structurally (Cheetham & Steitz, Science 1999; Durniak et al., Science 2008; Bandwar & Patel, JBC 2001), and both enzymes are routinely operated under saturating promoter conditions in IVT. Crucially, any differences in initiation efficiency, abortive cycling or open-complex stability between the two enzymes—whether intrinsic or modulated by modified NTPs—are convolved into the steady-state RNA yield and product-

quality readouts that we already report (Figs. 2 and 3). An additional, isolated measurement of promoter affinity would therefore not change which enzyme produces more, or cleaner, full-length transcript with a given modified nucleotide; that question is answered directly by the endpoint comparison we present.

3. Rate of modified-nucleotide incorporation and the choice of endpoint analysis

We agree that single-nucleotide-addition rate constants are an interesting biophysical parameter. We would, however, like to make three points explaining why such measurements are not necessary to support the claims of this manuscript.

First, single-turnover incorporation kinetics for modified nucleotides with T7 RNA polymerase have already been reported in the literature for the analogues most relevant to therapeutic mRNA, including pseudouridine, N1-methyl-pseudouridine and 5-methylcytidine (Milisavljević et al., ACS Omega 2024; Padilla & Sousa, NAR 2002). The general conclusion of those studies—that common base-modified UTP/CTP analogues are incorporated by T7 with elongation rates within the same order of magnitude as the canonical NTPs—is fully consistent with the yields and product distributions we observe. Repeating these measurements would therefore not change the interpretation of our data.

Second, for the comparison we set out to perform, the relevant figure of merit is not the elementary incorporation rate but the integrated outcome of an entire IVT reaction: full-length RNA yield, length homogeneity and the level of dsRNA by-product. These quantities depend on initiation, elongation, dissociation, abortive cycling, RNA re-binding and 3′-extension by the polymerase (Wu et al., Nucleic Acids Res. 2024; Mu et al., Front. Mol. Biosci. 2024), and they cannot be predicted from a single-step rate constant. Endpoint analysis under standardized IVT conditions is the field-standard readout used in the directed-evolution and process-development literature on T7 RNA polymerase variants for mRNA manufacturing (Dousis et al., Nat. Biotechnol. 2023; Wu et al., NAR 2024; the recently described M30 variant, PNAS Nexus 2026), precisely because it integrates these contributions in the regime that is therapeutically relevant.

Third, our experimental design specifically isolates the effect of polymerase identity: identical templates, NTP pools, buffer compositions, temperatures and reaction times are used for T7 and SP6 in side-by-side reactions. Within this design, differences in the apparent rate of incorporation of a given modified NTP manifest themselves as differences in the relative yield and quality of the transcript, both of which we quantify. Accordingly, we have added the following sentence to the section “Activity and substrate tolerance of T7 RNAP, SP6 RNAP and their mutants on a short template” of the revised manuscript: *“All reactions were carried out under matched conditions with identical templates downstream of the polymerase-specific promoter, identical NTP pools, buffer composition, temperature and reaction time, so that any differences in promoter recognition, initiation efficiency, abortive cycling or modified-nucleotide incorporation rate between T7 and SP6 RNAP are reflected directly in the integrated*

readouts of full-length transcript yield and length distribution. Endpoint analysis under such matched conditions has been established as the appropriate readout for comparing IVT performance, including by-product profiles, in studies of T7 RNAP variants aimed at therapeutic mRNA production [21, 26, 31, 37].

4. Which polymerase is better for which nucleotide?

This is in fact one of the central questions our manuscript answers. Figures 2 and 3 provide the side-by-side comparison of T7 and SP6 RNA polymerases across the panel of canonical and modified nucleotides examined, in terms of full-length transcript yield and dsRNA by-product formation. The endpoint nature of the experiment is, as discussed above, the appropriate readout for this question. Accordingly, we have made two changes to the revised manuscript. First, in the section “Activity and substrate tolerance of T7 RNAP, SP6 RNAP and their mutants on an extended template”, we replaced the original sentence *“While TapeStation analysis revealed overall lower RNA concentrations in reactions performed with SP6 RNAP F and FA, SP6 RNAP FA was the only polymerase capable of producing a clearly visible amount of full-length RNA under given conditions.”* with the more direct formulation *“Among all enzymes tested, SP6 RNAP FA was the only polymerase that produced a clearly resolved full-length 760 nt transcript with 2’□OMe□UTP, despite its overall lower total RNA concentration; this lower concentration is consistent with reduced abortive cycling rather than impaired full-length synthesis, as discussed below.”* Second, we have added the following short summary paragraph at the end of the same section so that the polymerase–nucleotide preferences are stated explicitly: *“Taken together, Fig 2 and Fig 3 define a clear polymerase-by-nucleotide preference profile across both template lengths. With unmodified UTP, both enzymes synthesize full-length transcripts, but T7 RNAP and its variants give consistently higher yields on the 760 nt template. With 2’-F-UTP, the F variants of both polymerases are the most active, with T7 RNAP F showing the highest acceptance of this modification. With the bulkier 2’-OMe-UTP, the polymerase ranking inverts: only SP6 RNAP variants produce detectable full-length 760 nt RNA, with the SP6 RNAP FA variant uniquely yielding a clearly resolved full-length band, whereas T7 RNAP variants are limited to truncated products under identical conditions. Thus, T7 RNAP F is the preferred enzyme for 2’-F-modified mRNA, while SP6 RNAP FA is the preferred enzyme for the incorporation of bulkier 2’-OMe modifications into long transcripts.”*

5. Use of a mixture of T7 and SP6 RNA polymerases

We thank the reviewer for raising this idea, which we agree is conceptually interesting. There are, however, well-defined mechanistic reasons why a mixed-polymerase experiment cannot serve the purpose of the present study.

Because the T7 and SP6 promoters are strictly orthogonal, a single template carries only one functional promoter and is transcribed by only one of the two enzymes; the second polymerase contributes only background non-specific or end-initiated transcription. Conversely, on a template bearing both promoters, T7 and SP6 elongation complexes

generate transcripts of identical sequence, so that the contribution of each enzyme to yield, modified-nucleotide incorporation and dsRNA by-product formation cannot be deconvolved without isotope labelling or promoter-specific tagging—experiments that constitute a separate study in their own right. A mixed-polymerase reaction therefore loses precisely the controlled, head-to-head comparison that is the central contribution of this work. Accordingly, we have added the following paragraph as the closing paragraph of the Conclusion of the revised manuscript: *"Building on the complementary substrate preferences uncovered here, namely T7 RNAP for 2'-F-modified RNA, SP6 RNAP FA for 2'-OMe-modified long RNA, and SP6 RNAP wt for low-dsRNA synthesis, a logical next step will be to explore whether these strengths can be combined within a single workflow. One avenue is mixed-polymerase IVT, in which T7 and SP6 RNAP are used in parallel reactions and the products are pooled, or, more ambitiously, in a single reaction where promoter orthogonality is exploited to assign distinct templates to each enzyme. Such studies will require dedicated experimental designs to deconvolve the contribution of each polymerase to yield, modified-nucleotide incorporation and dsRNA formation, for example, through promoter-specific sequence tags or differential isotopic labelling. Together with further structure-guided engineering of SP6 RNAP, such approaches may enable hybrid IVT platforms that combine the high yields of T7 RNAP with the favorable purity and modified-nucleotide tolerance demonstrated here for SP6 RNAP."*

We hope that these clarifications, together with the corresponding edits to the manuscript, address the reviewer's concerns and make clear why the additional experiments suggested, while interesting in their own right, are not required to substantiate the claims of the present comparative study.
